# Sustainability: a multifaceted important aspect of cancer care

**DOI:** 10.1038/s44276-023-00025-7

**Published:** 2024-03-08

**Authors:** Seamus O’Reilly, Catherine S. Weadick, Rachel J. Keogh

**Affiliations:** 1 Department of Medical Oncology, Cork University Hospital, Cork, Ireland; 2 Cancer Research @UCC, School of Medicine and Health, University College Cork, Cork, Ireland; 3 Cancer Trials Ireland, Royal College of Surgeons in Ireland, St Stephens Green, Dublin 2, Ireland; 4 Department of Medical Oncology, University Hospital Galway, Galway, Ireland

The COVID-19 pandemic will be the most significant global medical event in the lifetime of the majority of readers of this article. Its dramatic impact led to 19th century infection control and social distancing measures leading to lockdowns to prevent healthcare systems being overwhelmed until 20th century vaccination programmes were developed. The measures to sustain healthcare and society were a microcosm of the medical ecosystem. These included the acceleration of innovation in a crisis [1], with the integration of phase III trials and real-world data led to the successful impact of novel mRNA vaccines on COVID-19 mortality and the pragmatic RECOVERY trials [2–5]. However, it also exposed vulnerabilities and magnified inequalities, as reflected in higher COVID-19-related mortality rates in lower socio-economic groups [6–10] and ethnic minorities [11, 12], the impact of medical mistrust and misinformation [9, 13], and it will also be reflected in the delayed recovery in low- and middle-income countries post-pandemic, as predicted by the World Bank [14]. Global warming will triple the risk of similar pandemics as animal migration increases the risk of zoonotic illness [15, 16].

The pandemic highlighted the need for sustainability of cancer care to prevent a parallel pandemic, as the disruption and lack of availability of care has been a major concern [17–21]. Statistical modelling during the pandemic outlined that when cancer service disruption occurs for 6 months, mortality increases for a decade [22]. In Ireland, this disruption was compounded by the first ever cyberattack on a national health service at the end of the 3rd wave of the COVID-19 pandemic [19, 20]. Such concerns regarding the impact of disruption on mortality have been recently reinforced by a cross-sectional study of 1535 Syrian refugees cared for in haematology–oncology departments of eight university hospitals in the southern province of Turkey between January 1st 2011 and December 31st 2020 [23]. Real world 5 year survival rates of 17.5% in adults and 29.7% in children were observed. The 5-year survival rate for breast cancer was 37.8% in the cohort; in contrast, the contemporaneous 5 year survival in Ireland was 86.3% [24]. Late presentations, treatment abandonment and delayed diagnosis were responsible for these low survival rates. In adult patients with, and without, treatment abandonment, the survival rates were 9% and 26.6%, respectively.

These stark figures will be compounded by the transgenerational impact of associated parental deaths [25, 26]. They highlight that when cancer care is not sustainable, then the impact on patients and society is significant.

The most significant global health event in our lifetimes is also an opportunity for positive transformational change [27–30]. This is timely for cancer care where improvements have not been equitably implemented [31, 32], and the current era of therapeutic advances is compromised by the rising costs of anti-cancer agents which limits their relevance [33–35]. Innovation is declining at a time when it is needed, particularly in low- and middle-income countries where by 2040, 30 million new cancer cases and 16 million cancer-related deaths will be reported [35–37]. The societal impact will be magnified by the projected shortfall of 10 million healthcare workers globally, particularly in these countries [38].

Such needed transformational change can only occur if our existing systems are sustainable. By sustainable, we mean meeting the needs of the present without compromising the ability of future generations to meet their own needs. Sustainability in healthcare is multifactorial and includes workforce recruitment and retention, on encouraging and engaging with the next generation of researchers [39–42], on funding, and on environmental stability. In recognition, the editorial board of BJC Reports has made sustainability a North Star for the journal. This will be reflected in the diversity of the editorial board, by the integration of trainees into the peer review process, and by the promotion of articles related to sustainability in the journal. Our vision is that the journal will be a source of information and engagement on issues that impact delivery of sustainable cancer care, and that this will not be limited to articles on climate change, but also include those on trust, equity, funding, education and training – all of which are required to deliver cancer care to our communities. The sustainability section will also welcome solution based articles that highlight means to reduce the environmental footprint of cancer care, that improve equity in healthcare delivery, and address healthcare workforce wellbeing.

In this regard, we will be launching a new section in the journal on ‘Sustainability in Oncology’. The inaugural article by Professor Joan Schiller and a founder of the OUCH (Oncologists United for Climate and Health) (www.ouchforclimate.org) is published as the first article in the section [43, 44]. The advocacy she promotes is needed, at a time when climate change threatens health care provision in all countries, but particularly those in the global south [45]. There bioarchaeology studies have demonstrated that global warming will increase the likelihood of conflict [46], replicating the devastating cancer outcomes observed among Syrian refugees [23], and mirroring the inequalities that the COVID-19 pandemic has highlighted [7].

Professor Schiller’s article emphasises the place of our profession as ‘trusted messengers’ in our communities and the need to leverage that trust. Recently in the United States, the Joint Commission, a regulatory body involved in hospital accreditation relegated its hospital sustainability standards, originally proposed as mandatory to ‘optional’ in the face of industry objections [46]. If healthcare in the United States was a nation it would rank 13th globally related to emissions [47]. This relegation marked a missed opportunity to embed climate smartness in health care there, and to be an exemplar to other groups. It contrasts with efforts being made in the United Kingdom where the National Health Service ‘Delivering a Net Zero Health Service’ aims to reach net zero by 2030 [48]. It comes at a time of political push back on climate mitigation, and highlights the need for our healthcare community to be more engaged professionally and personally in this area [48, 49]. The article is a call to action to our community to address climate change, which increases cancer incidence, compromises cancer care, and is also directly contributed to by cancer care [50]. A recent survey of 4654 healthcare professionals has highlighted the personal, professional and societal barriers that prevent such advocacy and the potential for policy statements, guidance on work place sustainability and other measures to address them [51]. Such societal and workplace engagement is needed as we witness extreme climate events (wildfires, droughts, floods), compounded by the impact of pandemics (including COVID-19 and cholera), and the Russia Ukraine war, jeopardising sustainable healthcare delivery [52, 53]. During the COVID-19 pandemic unity of purpose characterised the response of healthcare professionals; we agree with Professor Schiller that the same unity of purpose and solidarity is needed now. The writer Jean Paul Sarte wrote that ‘Every word has consequences. Every silence, too’. We need to stop being silent.
